# Stress begets stress: the association of adverse childhood experiences with psychological distress in the presence of adult life stress

**DOI:** 10.1186/s12889-018-5767-0

**Published:** 2018-07-05

**Authors:** Mercy Manyema, Shane A. Norris, Linda M. Richter

**Affiliations:** 1 DST-NRF Center of Excellence in Human Development, University of the Witwatersrand, 1st Floor School of Public Health Building, Wits Education Campus, 7 York Road, Parktown, Johannesburg, 2193 South Africa; 2 MRC/Wits Developmental Pathways for Health Research Unit, Corner College & Clinic Road, Chris Hani Baragwanath Academic Hospital, University of the Witwatersrand, Johannesburg, South Africa

**Keywords:** Adverse childhood experiences, Psychological distress, Mental health, Stressful life events, Young adult, Birth to twenty plus

## Abstract

**Background:**

Adverse childhood experiences (ACES) have been linked to poor health and well-being outcomes, including poor mental health such as psychological distress. Both ACEs and psychological distress pose a significant public health burden, particularly in low to middle income countries. Contemporaneous stress events in adulthood may also impact psychological distress. The aims of this study were to describe the prevalence of ACEs and psychological distress and to assess the separate and cumulative effect of ACEs on psychological distress, while accounting for the effect of adult stress.

**Methods:**

In this cross-sectional study, we used retrospectively measured ACEs from a sample of 1223 young adults aged between 22 and 23 years (52% female) from the Birth to Twenty Plus Study. Psychological distress and adult life stress were measured with a six-month recall period. Hierarchical logistic regression was employed to assess the associations between the exposures and outcome.

**Results:**

Nearly 90% of the sample reported at least one ACE and 28% reported psychological distress. The median number of ACEs reported was three (range 0–11). After accounting for demographic and socio-economic factors, all ACEs were individually associated with psychological distress except for parental divorce and unemployment. The individual ACEs increased the odds of PD by between 1.42 and 2.79 times. Compared to participants experiencing no ACEs, those experiencing one to five ACEs were three times more likely to report psychological distress (AOR 3.2 95% CI: 1.83–5.63), while participants who experienced six or more ACEs had nearly eight times greater odds of reporting psychological distress (AOR 7.98 95% CI: 4.28–14.91). Interaction analysis showed that in the absence of adult life stress, the effect of low ACEs compared to high ACEs on PD was not significantly different.

**Discussion and conclusion:**

The prevalence of ACEs in this young adult population is high, similar to other studies in young adult populations. A significant direct association exists between ACEs and psychological distress. Adult life stress seems to be a mediator of this relationship. Interventions targeted at psychological distress should address both early life adversity and contemporary stress.

**Electronic supplementary material:**

The online version of this article (10.1186/s12889-018-5767-0) contains supplementary material, which is available to authorized users.

## Background

Adverse childhood experiences (ACEs) have been linked to a myriad of poor behavioral and health outcomes. ACEs as defined by the Kaiser-Permanente Study, also known as the ACE Study, include child abuse and neglect and growing up in dysfunctional households characterized by domestic violence, mental illness and incarceration of a household member, parental divorce or separation and household drug or alcohol abuse [1]. An increasing body of evidence points to a consistent link between ACEs and poor mental health in both children and adults. The ACE Study itself showed that the risk of suicide attempts increased two to five-fold with experiencing any ACE and an ACE score of four or more was associated with increased risk of attempted suicide, lifetime depressive disorders and poor mental health in general [2–4]. Strong dose-response relationships between ACEs and depressive symptoms, drug abuse and antisocial behavior as well as psychological distress (PD), perceived well-being and missed days of work have also been demonstrated [5, 6]. A South African study, though not using the full description of ACEs, found a significant association between childhood emotional neglect and adult depression, alcohol problems and suicidality [7].

A high proportion of children from different settings are exposed to ACEs. The ACE study reported a 60% prevalence of at least one ACE and 10% five or more ACEs [1, 8]. Nationally representative surveys conducted in England and Wales revealed that 46 and 47% of people, respectively, suffered at least one ACE and 8.3 and 14% respectively experienced four or more ACEs [9, 10]. In Brazil, 85% of an adolescent cohort reported experiencing at least one ACE in their lifetime [11]. According to Jewkes et al., 54.7 and 56.4% of rural South African women and men experience emotional abuse respectively, 41.6 and 39.6% experienced emotional neglect, and 39.1 and 16.7% experienced sexual abuse before the age of 18 [7].

Mental, neurological and substance use disorders constitute a significant global disease burden, with 10% of global disability-adjusted life years (DALYs) attributed to these disorders in 2010 [12, 13]. More than 1.1 billion people worldwide suffered from mental and substance use disorders in 2016 according to the Global Burden of Disease Study, constituting 18.6% of the total non-fatal disease burden [14]. In the South African Stress and Health (SASH) Study conducted between 2002 and 2004, the lifetime prevalence of major depression was 9.7, and 4.9% for the past year; 9.8 and 4.9% for mood disorders, and 15.8 and 8.1% for anxiety disorders respectively [15, 16]. Mental health remains neglected in many countries with a large gap existing between the burden caused and resources available to address mental health problems [17]. PD, which is defined as a “state of emotional suffering” characterized by depressive, anxiety as well as some somatic symptoms, is widely used as a marker of population mental health [18]. Evidence shows that it is positively associated with mental disorders and mental well-being [19].

The occurrence of stressful life events in adulthood increases the likelihood of experiencing psychological distress, as do race and availability of material resources, according to the SASH Study [20]. A complex interplay exists between early life stress, socioeconomic status and later life adversity. The stress sensitization theory has been posited as a possible mechanism through which early adversity and stress in later life and poor health are connected. It suggests that repeated stress early in life dysregulates stress response systems and lowers the threshold for reactivity and adaptive responses to subsequent stress. This increases the risk of mental health disorders in later life [16, 21]. In the same vein, Pearlin et al. put forward the theory of stress proliferation, a process through which stress begets stress, i.e. exposure to serious adversity in childhood increases the risk for later exposure to additional adversities [21, 22]. Childhood adversity can result in secondary stressors that cause harmful health consequences either together with or in place of the initial event, with some secondary stressors possibly being other adverse events [22].

Several other factors have been identified as correlates of poor mental health. The prevalence of major depression and PD has been found to be higher among females than males, among people with lower levels of education and among those aged 40 to 49 years [15, 23]. Increased risk of PD has been reported for unemployed and disabled persons as well as for those experiencing financial difficulties [23–25]. Some evidence suggests that household dysfunction ACEs are linked with the highest risk of mental disorders compared to abuse and neglect [26].

The relationship between ACEs and mental health is of marked public health importance. This association should however be examined in light of the several factors that may influence it, including adult stress and SES. The majority of literature on the epidemiology of ACEs and their impact on mental health come from high-income countries. The objectives of this study were: (1) to describe the prevalence of ACEs and PD in a young adult South African sample, (2) to examine the association between individual ACEs and PD and (3) to assess the cumulative effect of ACEs on PD, while accounting for the effect of adult stress on this relationship.

## Methods

### Data and study population

Data for this cross-sectional study were drawn from the Birth to Twenty Plus (Bt20+) Study. Named the Birth to Ten Study (Bt10) at its inception, the Bt20+ has been following up urban children and tracking their growth, health, well-being and educational progress from 1990 to date [27]. The study is based in Soweto, a densely populated suburb in the Greater Johannesburg Metropolitan area in South Africa, with an estimated population of about 1.5 million in 2017 [27, 28]. In the period between 23 April and 8 June 1990, 3273 singleton children born to women resident in Soweto-Johannesburg during the designated 7-week enrolment period and for a further 6 months post-partum were recruited into the study [27, 29]. More recruitment details are described elsewhere [27]. Of the initial cohort, 1636 were interviewed between 2012 and 2013 when the cohort was aged between 22 and 23 years and data for this analysis were drawn from this data collection wave.

### Measures

#### Psychological distress

The main outcome measure, psychological distress (PD), was assessed using the General Health Questionnaire 28 (GHQ-28), a screening tool developed to test the risk of developing psychiatric disorders [30]. It comprises 28 items arranged in four subscales: somatic symptoms; anxiety/insomnia; social dysfunction, and severe depression. Each item has four possible responses namely Not at all, No more than usual, Rather more than usual, and Much more than usual, coded 0 to 3. The recall period for the GHQ-28 is 6 months. The total score for each participant was computed with a possible score of 21 for each subscale and a global total of 84. In line with literature, a total score of 24 was considered to be the threshold for PD, creating a binary variable for PD [30]. The four subscales were also analysed separately as count variables. The internal validity of the GHQ-28 was tested using Cronbach’s alpha analysis. An alpha coefficient of 0.9013 was obtained for correlation between all the 28 items and values of 0.7649, 0.8541, 0.7608 and 0.8802 were obtained for the somatic, anxiety, social dysfunction depression subscales respectively. All the questionnaire items were therefore retained in the analysis.

#### Adverse childhood experiences

Using a questionnaire adapted from the ACE Study Questionnaire [31], ACEs were retrospectively reported at 22–23 years of age. Data on chronic illness, unemployment and parental death were also collected in addition to the original ten ACEs used in the ACE study, based on recommendations to include some experiences that may be prevalent and relevant in low-income settings [32]. Additional File 1 shows the questions used to assess ACEs. A point was allocated to each ACE exposure for which a participant answered yes to one or both questions, i.e. only one point could be allocated to each ACE.

ACEs were analyzed as single variables, or grouped into abuse (emotional, physical and sexual abuse), neglect (emotional and physical neglect) and household dysfunction (parental divorce, parental death, drug abuse in household, mentally ill household member, chronically ill household member, incarcerated household member, witnessing domestic violence and unemployment of caregiver) categories. An ACE score was computed by adding the number of ACEs to which each person was exposed. Three categorical variables were created based on the ACE score:

1. A binary variable to indicate the proportion of participants who had experienced at least one ACE before the age of 18 years.
2. A variable with five ACE score categories to indicate those who experienced no ACEs, one, two, three and four or more ACEs, to allow comparability with similar studies [1].
3. A variable was created to broadly divide the ACE score into three categories: no ACEs (score of 0), lower level of ACEs (score 1 to 5) and higher level of ACEs (score 6 to 13).

These three variations of the ACE score were created to gain a better understanding of the cumulative effect of ACEs on mental well-being, as well as to have results comparable to similar studies.

#### Stressful life events in adulthood

Data were collected on the participants’ experience of stressful life events during the preceding 6 months. Participants at age 22–23 years completed a questionnaire asking if the following had happened: injury of a household member due to violence, family member was a victim of crime, witnessing a violent crime, illness of a close family member, death of a close family member, living with someone with a serious disability in the family, drug or alcohol abuse in the household, fighting with or alienation from a close family member or neighbour, and incarceration of a family member. With each item assigned one point, a score of stressful life events experienced was computed and a categorical variable created with three categories: no experience (0), low levels (score of 1 to 3) and high levels of adult stress (score of 4 or more).

#### Socioeconomic and demographic factors

Previous work done on the Bt20+ data suggests that a wealth index is useful in adjusting regression models for SES [33]. At 22–23 years of age, participants were asked questions regarding ownership of thirteen household assets: television, car, washing machine, fridge, phone, radio, microwave, cell phone, DVD/Video player, DSTV (satellite television), computer, internet and medical aid /medical insurance. We assessed SES using a summative wealth index based on this list of assets. SES was analysed as a continuous variable with scores ranging between 1 and 13. The scores were normally distributed with the mean very close in value to the median.

The questionnaire also collected data on gender, age, current marital status, graduation from high school or completing matric, and current employment status.

### Statistical analysis

#### Descriptive statistics

Prevalence of ACEs and demographic characteristics were described by the presence or absence of PD, and chi-square and analysis of variance (ANOVA) tests used to test for significant differences between them. The Kruskal-Wallis test was used to assess the difference in the reporting of subscale symptoms among those reporting at least one ACE compared to those with no ACE. The distribution of the ACEs grouped into abuse, neglect and household dysfunction is also described.

#### Logistic regression: Effects of individual ACEs on PD

Logistic regression was used to determine the unadjusted and adjusted association between ACEs and PD as follows:

- The association between each individual ACE and PD was tested in univariate analysis as well as adjusted for demographic, SES and adult stress factors
- A model was created that included all individual ACE variables and the demographic factors to assess for overlapping effects between the ACES.
- Negative binomial regression was used to test for associations between ACEs and the different GHQ subscales and adult life stress.
- The association between ACEs and adult life stress were also tested in univariate and multivariate analysis

#### Hierarchical logistic regression

**Fig. 1:**
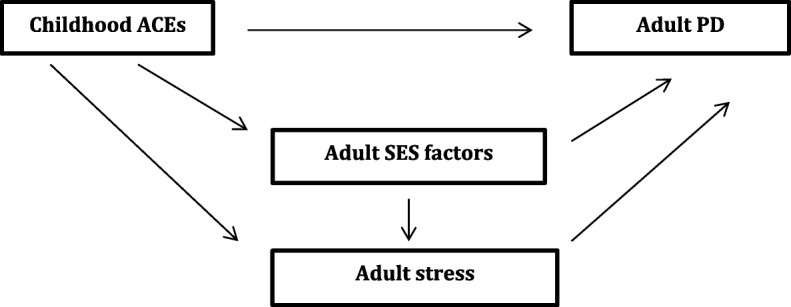
Conceptual framework for the hierarchical regression. ACEs – Adverse childhood events; PD-psychological distress; SES-socioeconomic status

- We used hierarchical regression to test the association between the cumulative ACE score variables and PD in order to assess the direct relationship, the potential multiple pathways linking them as well the cumulative effects of not only ACEs but also adult stressful life events. In step 1, we adjusted for demographic variables of age, gender and marital status. The second step added household SES, current employment and completion of high school. The adult life stress variable was added in the third step of the hierarchical regression.
- An interaction term between ACEs and adult life stress was also tested in separate models, the term being added to the model in the third step. Figure 1 shows the conceptual framework used in the hierarchical analysis.

The framework hypothesizes a direct association between ACEs and PD independent of other factors. However, ACEs may act through SES and adult stress to impact mental well-being. SES factors themselves may also have an effect on adult stress. Demographic factors, not shown in the framework are potential confounders that may be associated with both ACEs and PD.

#### Missing data

We performed descriptive statistics on participants with missing ACE and PD data to check if there were any significant differences in demographic characteristics.

## Results

### Descriptive analysis

Table 1 presents the demographic characteristics of the sample and the prevalence of ACEs and PD.

**Table 1:** Prevalence of ACEs and demographic characteristics of the sample

Variable	PD No n (%)	PD Yes n (%)	Total N (%)	*p*-value
Cumulative ACE Variables				
Any ACE				
No	131 (15)	16 (5)	147 (13)	
Yes	735 (85)	308 (95)	1043 (87)	< 0.001
ACE score categories				
None (0 ACEs)	131 (15)	16 (5)	147 (13)	
1 ACE	180 (21)	55 (17)	235 (20)	
2 ACEs	161 (19)	51 (16)	212 (18)	
3 ACEs	137 (16)	44 (14)	181 (15)	
4 or more ACEs	257 (30)	158 (49)	415 (35)	< 0.001
Level of ACEs				
None (0 ACEs)	131 (15)	16 (5)	147 (12)	
Low (1 to 5)	640 (74)	228 (70)	868 (73)	
High (6 or more)	95 (11)	80 (25)	175 (15)	< 0.001
Abuse				
No	731 (69)	220 (53)	951 (64)	
Yes	328 (31)	196 (47)	524 (36)	< 0.001
Neglect				
No	812 (74)	217 (50)	1029 (67)	
Yes	282 (26)	214 (50)	496 (33)	< 0.001
Household dysfunction				
No	169 (16)	29 (7)	198 (13)	
Yes	892 (84)	400 (93)	1292 (87)	< 0.001
Individual ACE Variables				
Emotional abuse				
No	812 (74)	256 (59)	1068 (70)	
Yes	282 (26)	175 (41)	457 (30)	< 0.001
Sexual abuse				
No	1032 (97)	385 (93)	1417 (96)	
Yes	31 (3)	27 (7)	58 (4)	0.001
Physical abuse				
No	997 (94)	369 (89)	1366 (92)	
Yes	68 (6)	44 (11)	112 (8)	0.005
Emotional neglect				
No	861 (78)	235 (54)	1096 (72)	
Yes	236 (22)	197 (46)	433 (28)	< 0.001
Physical neglect				
No	992 (91)	355 (82)	1347 (88)	
Yes	101 (9)	77 (18)	178 (12)	< 0.001
Domestic violence				
No	977 (90)	346 (80)	1323 (87)	
Yes	114 (10)	84 (20)	198 (13)	< 0.001
Parental divorce/separation				
No	534 (57)	179 (51)	713 (55)	
Yes	401 (43)	174 (49)	575 (45)	0.039
Parental death				
No	794 (73)	305 (70)	1099 (72)	
Yes	301 (27)	128 (30)	429 (28)	0.417
Substance abuse				
No	836 (76)	278 (64)	1114 (73)	
Yes	264 (24)	155 (36)	419 (27)	< 0.001
Mental illness				
No	1003 (91)	345 (80)	1348 (88)	
Yes	96 (9)	88 (20)	184 (12)	< 0.001
Imprisonment				
No	852 (78)	325 (75)	1177 (77)	
Yes	246 (22)	108 (25)	354 (23)	0.289
Chronic illness				
No	837 (76)	292 (68)	1129 (74)	
Yes	259 (24)	140 (32)	399 (26)	< 0.001
Unemployment				
No	644 (59)	222 (51)	866 (57)	
Yes	456 (41)	211 (49)	667 (43)	0.010
Age	23 (SD 0.6)	23 (SD 0.6)	23 (SD 0.6)	0.494
Marital status				
Single	538 (49)	228 (52)	766 (50)	
Marital relationship	567 (51)	207 (48)	774 (50)	0.188
Gender				
Male	594 (54)	150 (34)	744 (48)	
Female	516 (46)	288 (66)	804 (52)	< 0.001
SES	9.4 (SD 2.3)	8.9 (SD 2.3)	9.3 (SD 2.2)	0.002
Secondary school graduation				
No	414 (38)	185 (43)	599 (39)	
Yes	680 (62)	247 (57)	927 (61)	0.073
Currently employed				
No	450 (47)	219 (57)	669 (50)	
Yes	503 (53)	166 (43)	669 (50)	0.001
Stressful life events (previous 6 months)				
No	352 (33)	85 (20)	437 (29)	
Low	619 (58)	254 (61)	873 (59)	
High	99 (9)	77 (19)	176 (12)	< 0.001

The sample comprised 1223 participants with complete ACE data of whom 51% were women and 49% men. Nearly 90% of the sample reported experiencing at least one ACE, 35% experienced at least four and 15% experienced at least six ACEs. The most frequently reported ACEs were parental divorce or separation and parental unemployment (45 and 43% respectively), while the least frequently reported were sexual abuse (4%) and physical abuse (8%). Over a third of the participants reported experiencing some form of abuse, 33% reported experiencing physical or emotional neglect and over 90% reported household dysfunction. The proportion of participants with PD was 28%, with 66% of these being women. Approximately 50% of those who had PD reported experiencing at least four ACEs, compared to 30% of those who had no PD, and 25% reported six or more ACEs, compared to 11% of those who did not have PD. Of the demographic factors, there was no significant difference in education and marital status between those who reported PD and those who did not.

#### Missing data

Nearly 2% of males and 1.3% of females did not have complete data for both ACEs and PD. Twenty-one percent of males and 23% of the females did not have complete ACE data, while 1.6 and 2.4% did not have complete PD data respectively and were thus (by default) not included in the regression models.

#### Reporting of individual GHQ subscales

Presented in Additional File 2 are histograms that show the number of participants reporting each of the four different GHQ subscales in relation to their experience of at least one ACE. The median scores for the somatic, anxiety/insomnia, dysfunction and depression subscales were 4 (range 0–19), 4 (range 0–21), 6 (range 0–21) and 0 (range 0–21) respectively. The median scores for the subscales for participants experiencing at least one ACE compared to no ACEs were: 4 and 2 for somatic, 4 and 2 for anxiety/insomnia, 6 and 5.5 for dysfunction and 1 and 0 for depression respectively. The Kruskal-Wallis analysis showed that there was a significant difference in reporting somatic, anxiety/insomnia and depression symptoms between those who experienced at least one ACE and those who experienced no ACEs (*p*-value < 0.05 for all three).

### Regression analyses

#### Effects of ACEs on adult life stress

Additional File 3 shows the separate and cumulative effects of ACEs on adult life stress. Compared to those who experienced no ACEs and low levels of ACEs, participants who reported high ACE levels had nearly twelve times greater odds of experiencing high levels of adult stress. All the demographic factors were not significantly associated with the experience of adult life stress. Parental death and sexual abuse were not significantly associated with adult life stress both in unadjusted and adjusted models.

#### Separate effects of ACEs on PD

The separate effects of ACEs on PD are presented in Table 2. Parental death and incarceration of household member were not associated with PD. After adjusting for demographic factors, parental divorce/separation and parental unemployment become statistically non-significant, pointing to possible confounding and/or mediation. In the model including all individual ACEs, the neglect ACEs and living with mental illness in the household remained statistically significantly increased the odds of PD, together with marital status, gender and adult life stress. Gender remained associated with PD in all adjusted models, with no evidence of reduction of effect size.

**Table 2:** Effects of individual ACEs on PD

Variable	Crude OR (95% CI)	Adjusted OR (95% CI) Individual ACEs^1^	Adjusted OR (95% CI) All individual ACEs in combined model^2^
Emotional abuse			
No	Ref	Ref	Ref
Yes	**1.96 (1.56–2.49)**	**1.93 (1.46–2.55)**	1.10 (0.75–1.63)
Sexual abuse			
No	Ref	Ref	Ref
Yes	**2.33 (1.38–3.96)**	**2.26 (1.20–4.25)**	1.78 (0.86–3.66)
Physical abuse			
No	Ref	Ref	Ref
Yes	**1.75 (1.17–2.60)**	**1.87 (1.20–2.93)**	1.14 (0.67–1.94)
Emotional neglect			
No	Ref	Ref	Ref
Yes	**3.05 (2.41–3.88)**	**2.79 (2.10–3.70)**	**1.99 (1.34–2.93)**
Physical neglect			
No	Ref	Ref	Ref
Yes	**2.13 (1.55–2.93)**	**2.24 (1.52–3.31)**	**1.64 (1.00–2.69)**
Domestic violence			
No	Ref	Ref	Ref
Yes	**2.08 (1.53–2.83)**	**1.91 (1.35–2.72)**	1.22 (0.78–1.90)
Parental divorce/separation			
No	Ref	Ref	Ref
Yes	**1.29 (1.01–1.65)**	1.13 (0.85–1.51)	0.96 (0.70–1.31)
Parental death			
No	Ref	Ref	Ref
Yes	1.11 (0.87–1.41)	1.26 (0.95–1.67)	1.15 (0.81–1.62)
Substance abuse			
No	Ref	Ref	Ref
Yes	**1.77 (1.39–2.25)**	**1.63 (1.22–2.17)**	1.24 (0.86–1.80)
Mental illness			
No	Ref	Ref	Ref
Yes	**2.66 (1.95–3.65)**	**2.72 (1.89–3.91)**	**2.25 (1.45–3.51)**
Incarceration			
No	Ref	Ref	Ref
Yes	1.15 (0.89–1.98)	1.12 (0.83–1.52)	0.79 (0.54–1.16)
Chronic illness			
No	Ref	Ref	Ref
Yes	1**.55 (1.21–1.98)**	**1.42 (1.07–1.89)**	1.07 (0.75–1.52)
Unemployment			
No	Ref	Ref	Ref
Yes	**1.34 (1.07–1.68)**	1.16 (0.89–1.51)	0.82 (0.59–1.15)
Marital status			
Single	Ref		Ref
Relationship	0.86 (0.69–1.08)		**0.64 (0.50–0.87)**
Gender			
Male	Ref		Ref
Female	**2.21 (1.76–2.78)**		**2.73 (1.98–3.77)**
SES	**0.91 (0.87–0.96)**		**0.90 (0.84–0.97)**
Completed matric			
No	Ref		Ref
Yes	**0.81 (0.65–1.02)**		0.84 (0.60–1.18)
Currently employed			
No	Ref		Ref
Yes	**0.68 (0.53–0.86)**		0.79 (0.58–1.08)
Stressful events			
No	Ref		Ref
Low	**1.70 (1.28–2.24)**		**1.67 (1.14–2.45)**
High	**3.22 (2.20–4.71)**		**1.80 (1.05–3.08)**

In unadjusted and adjusted analysis: **bold** = *p* < 0.05; ^1^ Each individual ACE entered as an exposure adjusted for demographic factors; all demographic factors except completing high school were significant at *p* < 0.05; ^2^All individual ACEs placed in one multivariate model, also adjusted for demographic factors

#### Association between ACEs and the separate PD subscales

The associations between the individual subscales and PD are shown in Additional File 4. The social dysfunction subscale was not statistically significantly associated with any of the cumulative ACE score variables, except experiencing at least six ACEs which was significant only in the univariate analysis. Of the demographic variables, household stress and gender significantly increased the relative risk of a high score in all four subscales. The depression subscale generally showed greater effect sizes compared to the other three subscales. A dose-response relationship is apparent in the three subscales that achieved statistical significance.

#### Hierarchical logistic regression

Table 3 shows the adjusted hierarchical regression analyses for the relationship between ACEs and PD, with the ACE score variable depicting three levels of ACEs: no ACEs, low level of ACES (1 to 5) and high level of ACEs (6 or more). Additional File 5 presents the same analyses using the binary ACE score variable.

**Table 3:** Hierarchical regression results: ACEs as a three-category exposure

	Model 1	Model 2	Model 3	Model 4 (with interaction)
Chi^2^ Δ	**103.3** ^**a**^	**110.0**	**123.3**	**129.6**
Level of ACEs				
None (0 ACEs)	Ref	Ref	Ref	Ref
Low (1 to 5)	**3.21 (1.83–5.63)**	**3.48 (1.81–6.67)**	**2.97 (1.53–5.72)**	2.49 (0.92–6.72)
High (6 or more)	**7.98 (4.28–14.91)**	**8.57 (4.19–17.53)**	**6.54 (3.14–13.63)**	**7.67 (1.90–30.94)**
Level of ACEs				
No stressful life events#No ACEs				Ref
Low stress#Low ACES				2.02 (0.47–8.59)
Low stress#High ACEs				1.33 (0.22–7.95)
High stress#Low ACEs				**0.10 (0.009–0.98)**
High stress#High ACEs				**0.08 (0.006–0.95)**
Age	1.10 (0.85–1.40)	1.09 (0.84–1.44)	1.09 (0.84–1.45)	1.09 (0.83–1.44)
Gender				
Male	Ref	Ref	Ref	Ref
Female	**2.61 (1.97–3.45)**	**2.67 (1.97–3.63)**	**2.71 (2.00–3.71)**	**2.77 (2.03–3.79)**
Marital status				
Single	Ref	Ref	Ref	Ref
Relationship	**0.70 (0.53–0.93)**	**0.68 (0.50–0.92)**	**0.67 (0.49–0.92)**	**0.66 (0.49–0.91)**
SES		**0.91 (0.85–0.98)**	**0.91 (0.84–0.98)**	**0.91 (0.84–0.98)**
Completed matric				
No		Ref	Ref	Ref
Yes		0.79 (0.57–1.09)	0.81 (0.58–1.12)	0.81 (0.58–1.13)
Employment				
No		Ref	Ref	Ref
Yes		0.79 (0.59–1.07)	0.80 (0.59–1.09)	0.80 (0.59–1.08)
Adult stress				
None			Ref	Ref
Low			**1.84 (1.26–2.68)**	0.99 (0.25–3.98)
High			**2.25 (1.35–3.74)**	**21.92 (2.35–204.86)**

^a^**Bold:** Likelihood ratio chi-square test significant at p < 0.05; ^#^**Bold OR:** significant at *p* < 0.05; ^Interaction terms

The full regression models for the two ACE score exposures achieved significance. The likelihood ratio chi-square statistic showed statistically significant improvement between the null model and tested model after each step in the hierarchical modelling. The effect of ACEs remained statistically significant even after adjusting for demographic, SES and adult life events stress factors. A dose-response association is noted between the ACE score and PD. The addition of the demographic and SES factors increased the effect size of the ACE score to levels higher than the unadjusted values, but adult stress brought the ORs down in all the three full models to levels lower than or similar to unadjusted. Being married was not associated with PD in the unadjusted model but was significant when entered into the model with gender. The effect size of gender on PD increased at each subsequent level of the model.

In the models with the interaction terms, it is apparent that high levels of adult life stress increase the likelihood of PD by over 20 times compared to no stress, in the absence of ACEs, while low levels of stress have a statistically non-significant association. Having experienced at least one ACE was significantly associated with PD even in the absence of adult life event stress (AOR 2.74, 95% CI 1.03–7.36). When the three category ACE score variable was used as the main exposure in Table 3, high levels of ACEs in the absence of adult stress increased the odds of PD by nearly 8 times. A closer look at the interaction terms shows that there was no significant difference in the effect of the level of ACEs on PD in the presence of low stress, i.e. the impact of one to five ACEs as well as experiencing six or more ACEs on PD did not differ significantly between those with low stress compared to those with no adult stress. However, both low and high levels of ACEs had a significantly different effect in individuals with high adult stress compared to those with no adult stress.

## Discussion

In this sample of young South African adults, 87% reported at least one ACE and 35% reported four or more ACEs. Nearly a third of the population reported signs of PD, of which 66% were women. Individual ACEs increased the odds of reporting PD by between 1.42 (chronic illness) and 2.79 (emotional neglect) times, after adjusting for demographic variables. Including all the ACEs in one model attenuated the effect of most ACEs except emotional and physical neglect and mental illness in the household. A significant direct association was observed between ACEs and PD, and a dose-response effect was apparent. A significant dose-response association was observed between ACE levels and adult stress levels with participants who reported experiencing six or more ACEs being 11 times more likely to experience high levels of adult stress (AOR 11.22**;** 95% CI 7.1–17.8) compared to those who reported zero and one to five ACEs.

Our ACE prevalence levels are similar to those of the Kaiser-Permanente ACE study where over 60% of the population had experienced at least one ACE [1]. However they are more consistent with a later study that used an expanded version of the ACE list and found that over 80% of the participants reported at least one ACE [34]. Although the additional ACEs included in our analysis: parental death, parental unemployment and chronic illness in the household were among the most prevalent, their effect became non-significant in the model combining all individual ACEs. This may mean that their effect is manifest through other ACEs or that they act as moderating factors. As ACE research is widely used to advocate for the protection of and greater investment in the early childhood years, it is important for the ACE indices to be broadly focused in order to capture a diversity of experiences that may impact life-long health and wellbeing [32, 34].

The higher prevalence of PD among women compared to men is consistent with results from others studies [16, 35], although some studies have found no gender differentials in the prevalence of poor mental health [6]. Compared to men, women who experienced at least one ACE were two and half times more likely to report PD, an effect that increased with the inclusion of SES and adult stress in the model. Greater odds of women having PD given the experience of ACEs compared to men have been reported in other studies, including in South Africa [36, 37].

The effect of most single ACEs on PD attenuated and became statistically not significant after accounting for other ACEs and demographic and SES factors. These results imply that the effect of some ACEs may only manifest through other exposures and that ACEs often co-occur and are highly interrelated. In this sample, physical and emotional neglect and mental illness in the household seem to be the most foreboding individual ACE exposures impacting mental well-being independently of other ACEs, adult stress, SES and demographic factors. A dose-response effect was observed between the ACE score and PD, even after accounting for adult stress, as well as demographic and SES factors. This shows an unmediated, direct effect of ACEs on PD, and that this effect increases with exposure to a greater number of ACEs. This finding is in line with a growing body of evidence that shows that early life adversity can disrupt not only brain structure and functioning, but also dysregulate other systems resulting in low stress thresholds that persist throughout life and increase the risk of stress-related disease or disorders [38, 39]. This direct effect is however not the only effect of ACEs on PD. The hierarchical modelling showed increasing effects of ACEs with the addition of demographic and SES factors to the models. This may be due to exacerbating moderation effects of these factors on the association between ACEs and PD.

The magnitude of the dose-response association between ACEs and adult life stress did not decrease after adjusting for demographic factors signifying a significant direct relationship between the two. Adult stressors may possibly be mediators of the association between ACEs and PD, demonstrated by the reduction of the effect of ACEs on PD after the addition of adult stress to the model. This means that participants exposed to ACEs may be more likely to show greater distress in the presence of adult stressors compared to those not exposed to ACEs, and that comparatively lower levels of stress may trigger distress [16]. The interaction analysis showed that in individuals that experienced high levels of adult stress, experiencing five ACEs or less, and experiencing six ACEs or more had significantly different effects on PD compared to experiencing no ACEs. Additionally, low ACE levels did not have a significant association with PD in the absence of adult stress. High levels of adult stress therefore significantly increased the likelihood of those who had a history of ACEs, especially cumulatively high levels of ACEs, also reporting PD. Although the results also suggest that adult stress on its own has a greater impact on PD than do ACEs, these two exposures possibly interact in several possible ways that have been suggested in previous research.

The interaction between ACEs and adult stressful events is admittedly complex, with ACEs possibly exacerbating the effect of subsequent stress on PD, and adult stress possibly mediating the relationship between ACEs and PD. These pathways are not fully elucidated here but our results are congruent with current knowledge. Evidence shows that exposure to ACEs can trigger neurophysiological sensitivity and erode the stress reactive and adaptive threshold thus stimulating dysfunctional coping strategies [22, 40]. Important psychosocial resources are undermined, resulting in lower levels of perceived social support and poorer perceptions of the self [40]. These shortfalls have negative mediating influences on positive subsequent development and physical, psychological, and social health outcomes in adulthood [40]. In addition to this heightened sensitivity, those who are exposed to ACEs may be at an increased risk of experiencing adult stress. The initial exposure to childhood stressors can lead to secondary effects through the inability to form and sustain healthy relationships, poor academic achievement and making decisions that leave individuals vulnerable to subsequent stressors like those investigated in this study [21]. Individuals exposed to childhood stress are hypothesized to have limited buffering of these secondary stressors compared to individuals without prior significant adversity exposure [21].

An important link between early life and adulthood stress, demonstrated in part through the hierarchical analysis, is through socio-economic status. Individuals exposed to ACEs are more likely to attain lower levels of education which lead to financial insecurity that can increase the risk of personal and family conflict, homelessness and unemployment [5]. Consequent to these adult adversities is the instability of social resources and reduced economic resources available to obtain professional help and maintain healthy habits [5, 41].

The Parental Acceptance–Rejection Theory (PARTheory) may be applied to explain the consistent link between neglect and PD in this sample. According to the theory, perceived parental acceptance or rejection affects psychological adjustment in childhood. In addition, when parental rejection that occurred in childhood is recollected later in life, it is likely to be associated with the same form of psychological maladjustment in adulthood [42, 43]. Parental acceptance refers to warmth, affection, love, care, comfort, support and nurturance, while parental rejection refers to the absence or withdrawal of warmth, affection, or love by parents towards their children [44]. An individual’s emotional security is partly dependent on the amount of warmth he or she receives from parents as a child and how he or she perceives parents’ warmth. Lack of experience of warmth may lead to low self-esteem, negative mental representations of self and others, anger, unresponsiveness, sadness, and emotional instability [45]. Those who report being neglected would be at an increased direct risk of PD in adulthood, apart from the influence of other factors. It is also possible that other forms of adversity such as physical and sexual abuse are perceived by the child as lack of love, comfort and warmth and have the same effect as neglect on the child’s and subsequently the adult’s psychological well-being.

The GHQ subscales are constructed to each represent common elements of symptomatology [46]. Our analysis therefore sought to assess the association of ACEs with each set of reported symptoms. Three subscales showed results in the expected directions but the social dysfunction subscale was not significantly associated with any of the cumulative ACE variables except high level of ACEs (unadjusted OR 1.16 (95% CI: 1.01–1.33). The items in the social dysfunction scale may not all be appropriate for our sample. However, women were more likely to report social dysfunction compared to men, those who were married and those who had jobs were less likely to report dysfunction compared to those who were single and unemployed respectively. These socio-demographic factors may have modified the association of ACEs with this subscale.

### Strengths and limitations

Our study utilized data from a long-running cohort with well-established data collection and management methods. Although a birth cohort is not a nationally representative sample two decades later, the data enable us to assess the presence of ACEs in a middle income population and test its association with poor mental well-being in adulthood. The experience of ACEs was reported retrospectively at 22 years of age, which may be subject to recall bias. The role of social coping resources in the association between ACEs and PD has been alluded to previously but we were not able to assess this here [47]. The missing observations may potentially affect the estimations as well but it may either be to under or over estimate.

## Conclusion and future implications

The experience of ACEs in this population compares with that found in other populations and points to the need for a more holistic approach in dealing with childhood adversity that includes identifying other forms of maltreatment and neglect when one adversity is reported. Treatment of mental illness and promotion of mental well-being should not only focus on contemporaneous events but also on possible childhood maltreatment and adversity.

Of all animal species, human offspring are dependent on their parents for the longest time. This means that any efforts to support healthy early childhood growth should necessarily include parental support and education. Governments and communities need to work together to support healthy family relationships and support parents in their efforts to raise their children. Effective and collective interventions are necessary in cases of adversity, abuse and violence in the household. Our study shows that this will contribute to improved mental wellbeing and other evidence suggests that it may also prevent other diseases [4, 10].

Early childhood development (ECD) is the foundation for sustainable development. Building a strong beginning for healthy development in the early years of life is essential for individual well-being, economic productivity and harmonious societies around the world [38, 48]. Every child, no matter where they live needs the best start in life in order for them to reach their full developmental potential. For the first time in the history of global development, ECD is a major part of the global development goals both directly in lifelong learning and embedded in several other goals. It is therefore time for governments, civil society, businesses, communities and individuals to work together to ensure that the importance of ECD takes center stage and protecting children from adversity is one of the key action points. The current analysis is useful in showing the extent of the problem, albeit in part, and provides a more comprehensive view than studying ACEs as single exposures.

## Additional files

Additional file 1: Questionnaire items used to assess the experience of ACEs. The table presents the questionnaire items derived from the WHO ACE-IQ used to measure the exposure to ACEs. (DOCX 15 kb)

Additional file 2: Number of participants reporting each of the GHQ subscales stratified by their experience of at least one ACE. The graphs present in each quadrant the participants who reported each of the four GHQ subscales, those who experienced no ACEs on the left of each quadrant and those who experienced at least one ACE on the right. (PDF 177 kb)

Additional file 3: Separate and cumulative effects of ACEs on adult life stress. The table presents the regression results of ACEs on adult life stress. (DOCX 14 kb)

Additional file 4: Association between ACEs and the different PD subscales. This table presents the associations between ACEs and the four GHQ subscales, obtained using negative binomial regression. (DOCX 15 kb)

Additional file 5: Hierarchical regression results: ACEs as binary exposure. Presented here are the adjusted hierarchical regression results using ACE as a binary exposure, including the interaction analysis. (DOCX 14 kb)

## Abbreviations

ACE: adverse childhood experiences
PD: psychological distress
SES: socioeconomic status
DALY: disability-adjusted life years
SASH: South African Stress and Health Study
YLD: years lived with disability
Bt20: Birth to Twenty Plus Study
Bt10: Birth to Ten Study
GHQ-28: General Health Questionnaire 28
DSTV: satellite television
ANOVA: analysis of variance
AOR: adjusted odds ratio
PARTheory: parental acceptance-rejection theory
