# Supplementary material for: Stress begets stress: the association of adverse childhood experiences with psychological distress in the presence of adult life stress
Source: BMC Public Health. 2018 Jul 5;18:835. doi: 10.1186/s12889-018-5767-0 (PMC6034311; doi:10.1186/s12889-018-5767-0)
Supplement: Supplementary file 1 — Questionnaire items used to assess the experience of ACEs. The table presents the questionnaire items derived from the WHO ACE-IQ used to measure the exposure to ACEs. (DOCX 15 kb) [file 12889_2018_5767_MOESM1_ESM.docx]

Questionnaire items used to assess the experience of ACEs

| **ACE** | **Question** | **Yes** | **No** |
| --- | --- | --- | --- |
| **Physical abuse** | Did a parent or other adult in the household **often or very often**…  Push, grab, slap, or throw something at you?  **OR**  **Ever** hit you so hard that you had marks or were injured? |  |  |
| **Sexual abuse** | Did an adult or person at least 5 years older than you **ever**…  Touch or fondle you or have you touch their body in a sexual way?  **OR**  Attempt or actually have oral, anal, or vaginal intercourse with you? |  |  |
| **Emotional abuse** | Did a parent or other adult in the household **often or very often**…  Swear at you, insult you, put you down, or humiliate you?  **OR**  Act in a way that made you afraid that you might be physically hurt? |  |  |
| **Emotional neglect** | Did you **often or very often** feel that …  No one in your family loved you or thought you were important or special?  **OR**  Your family didn’t look out for each other, feel close to each other, or support each other? |  |  |
| **Physical neglect** | Did you **often or very often** feel that …  You didn’t have enough to eat, had to wear dirty clothes, and had no one to protect you?  **OR**  Your parents were too drunk or high to take care of you or take you to the doctor if you needed it? |  |  |
| **Parental divorce** | Were your parents **ever** separated or divorced? |  |  |
| **Witnessing domestic violence** | Was your mother or stepmother:  **Often or very often** pushed, grabbed, slapped, or had something thrown at her?  **OR**  **Sometimes, often, or very often** kicked, bitten, hit with a fist, or hit with something hard?  **OR**  **Ever** repeatedly hit at least a few minutes or threatened with a gun or knife? |  |  |
| **Alcohol and/or drug abuse in the household** | Did you live with anyone who was a problem drinker or alcoholic or who used street drugs? |  |  |
| **Mental illness in the household** | Was a household member depressed or mentally ill, or did a household member attempt suicide? |  |  |
| **Imprisonment of household member** | Did a household member go to prison? |  |  |
| **Chronic illness (other than mental illness) in the household** | Was there anyone in your household who was chronically ill when you were a child? |  |  |
| **Unemployment of parent/caregiver** | Was one or more of your parents/caregivers mostly unemployed during your childhood because they could not get a job? |  |  |
| **Death of parent/caregiver** | Did either of your parents/caregivers pass away before you turned 18? |  |  |
