# Supplementary material for: Stress begets stress: the association of adverse childhood experiences with psychological distress in the presence of adult life stress
Source: BMC Public Health. 2018 Jul 5;18:835. doi: 10.1186/s12889-018-5767-0 (PMC6034311; doi:10.1186/s12889-018-5767-0)
Supplement: Supplementary file 2 — Number of participants reporting each of the GHQ subscales stratified by their experience of at least one ACE. The graphs present in each quadrant the participants who reported each of the four GHQ subscales, those who experienced no ACEs on the left of each quadrant and those who experienced at least one ACE on the right. (PDF 177 kb) [file 12889_2018_5767_MOESM2_ESM.pdf]

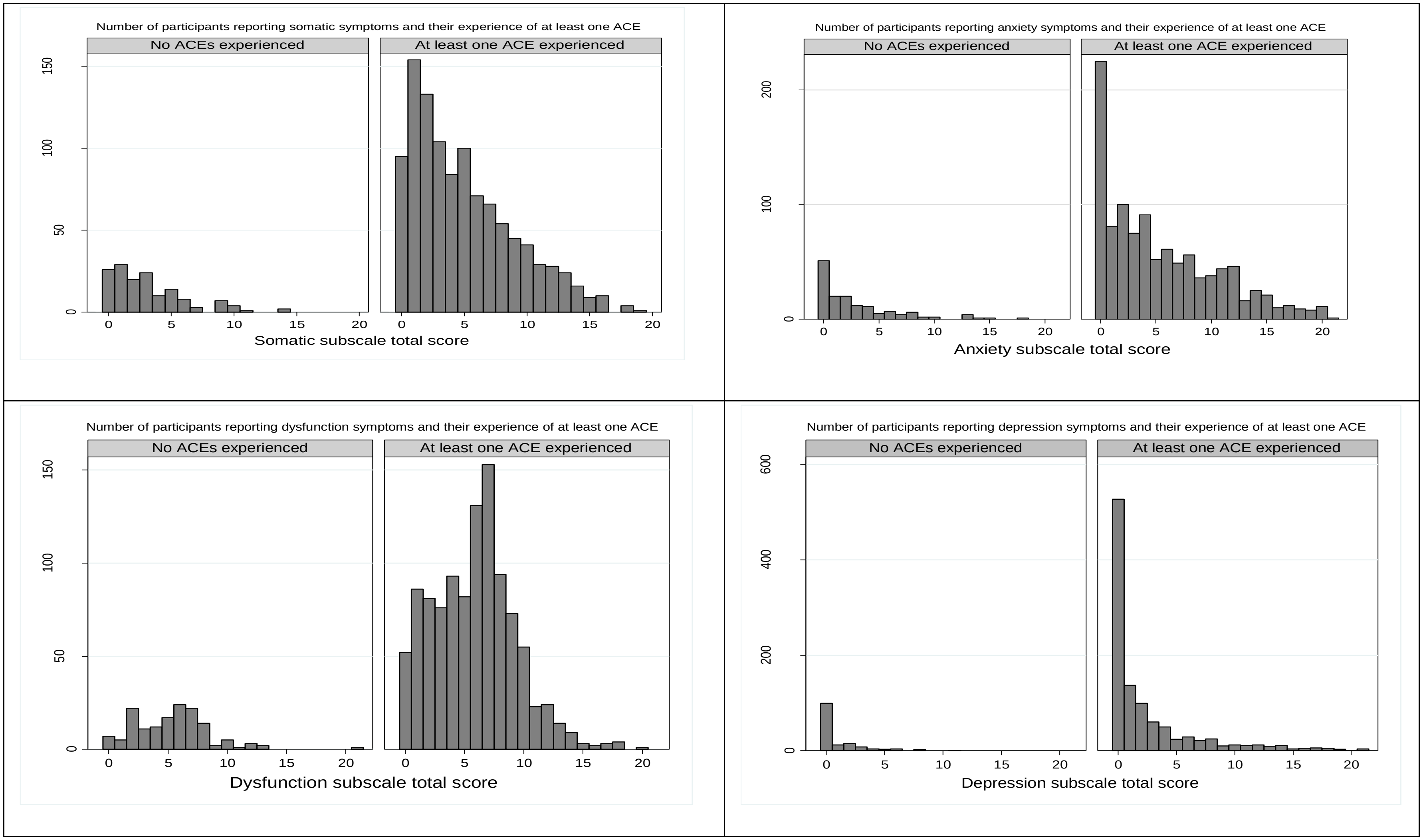

Number of participants reporting each of the GHQ subscales stratified by their experience of at least one ACE. In each quadrant are represented the participants who reported each of the four GHQ subscales, those who experienced no ACEs on the left of each quadrant and those who experienced at least one ACE on the right.
