# Supplementary material for: Stress begets stress: the association of adverse childhood experiences with psychological distress in the presence of adult life stress
Source: BMC Public Health. 2018 Jul 5;18:835. doi: 10.1186/s12889-018-5767-0 (PMC6034311; doi:10.1186/s12889-018-5767-0)
Supplement: Supplementary file 3 — Separate and cumulative effects of ACEs on adult life stress. The table presents the regression results of ACEs on adult life stress. (DOCX 14 kb) [file 12889_2018_5767_MOESM3_ESM.docx]

**Separate and cumulative effects of ACEs on adult life stress**

| **Variable** | **Crude OR**  **(95% CI)** | **Adjusted OR (95% CI)**  **Individual ACEs^** |
| --- | --- | --- |
| **Any ACE**  **No**  **Yes** | Ref  **3.70 (2.63-5.22)** | Ref  **3.60 (2.55 – 5.08)** |
| **Level of ACEs**  **None**  **Low**  **High** | Ref  **3.11 (2.20-4.40)**  **11.48 (7.28-18.11)** | Ref  **3.03 (2.14 – 4.30)**  **11.22 (7.08 – 17.75)** |
| **Emotional abuse**  **No**  **Yes** | Ref  **2.97 (2.36 – 3.75)** | Ref  **2.93 (2.32 – 3.71)** |
| **Sexual abuse**  **No**  **Yes** | Ref  1.26 (0.75 – 2.13) | Ref  1.21 (0.70 – 2.07) |
| **Physical abuse**  **No**  **Yes** | Ref  **1.82 (1.25 – 2.67)** | Ref  **1.85 (1.26 – 2.70)** |
| **Emotional neglect**  **No**  **Yes** | Ref  **2.90 (2.29 – 3.67)** | Ref  **2.82 (2.22 – 3.59)** |
| **Physical neglect**  **No**  **Yes** | Ref  **2.48 (1.81 – 3.39)** | Ref  **2.20 (1.59 – 3.04)** |
| **Domestic violence**  **No**  **Yes** | Ref  **1.79 (1.31 – 2.43)** | Ref  **1.76 (1.30 – 2.40)** |
| **Parental divorce/separation**  **No**  **Yes** | Ref  **1.44 (1.15 – 1.79)** | Ref  **1.36 (1.08 – 1.70)** |
| **Parental death**  **No**  **Yes** | Ref  1.12 (0.90 – 1.40) | Ref  1.08 (0.86 – 1.36) |
| **Substance abuse**  **No**  **Yes** | Ref  **3.49 (2.74 – 4.45)** | Ref  **3.37 (2.63 – 4.32)** |
| **Mental illness**  **No**  **Yes** | Ref  **3.78 (2.75 – 5.19)** | Ref  **3.63 (2.63 – 5.01)** |
| **Incarceration**  **No**  **Yes** | Ref  **2.60 (1.62 – 2.60)** | Ref  **2.45 (1.90 – 3.16)** |
| **Chronic illness**  **No**  **Yes** | Ref  **2.05 (1.62 – 2.60)** | Ref  **2.03 (1.60 – 2.57)** |
| **Unemployment**  **No**  **Yes** | Ref  **1.86 (1.51 – 2.28)** | Ref  **1.73 (1.40 – 2.14)** |

In unadjusted and adjusted analysis: **bold**=p<0.05; **^** Each individual ACE entered as an exposure adjusted for demographic factors**;**
