# Supplementary material for: Stress begets stress: the association of adverse childhood experiences with psychological distress in the presence of adult life stress
Source: BMC Public Health. 2018 Jul 5;18:835. doi: 10.1186/s12889-018-5767-0 (PMC6034311; doi:10.1186/s12889-018-5767-0)
Supplement: Supplementary file 4 — Association between ACEs and the different PD subscales. This table presents the associations between ACEs and the four GHQ subscales, obtained using negative binomial regression. (DOCX 15 kb) [file 12889_2018_5767_MOESM4_ESM.docx]

**Association between ACEs and the different PD subscales**

| **Variable** | **Crude IRR** | **Adjusted IRR** |
| --- | --- | --- |
| ***Somatic subscale*** | | |
| **Any ACE**  **No**  **Yes** | Ref  **1.60 (1.35-1.87)** | Ref  **1.46 (1.23-1.74)** **^Gender, household stress^** |
| **ACE score categories**  **None**  **1 ACE**  **2 ACEs**  **3 ACEs**  **4 or more** | Ref  **1.44 (1.19-1.73)**  **1.39 (1.15-1.69)**  **1.49 (1.23-1.81)**  **1.80 (1.52-2.13)** | Ref  **1.31 (1.07-1.60) ^Gender, household stress^**  **1.35 (1.10-1.66)**  **1.42 (1.15-1.76)**  **1.70 (1.40-2.06)** |
| **Level of ACEs**  **None**  **Low**  **High** | Ref  **1.50 (1.28-1.76)**  **1.98 (1.64-2.40)** | Ref  **1.41 (1.18-1.68)** **^Gender, household stress^**  **1.84 (1.48-2.28)** |
| ***Anxiety/insomnia subscale*** | | |
| **Any ACE**  **No**  **Yes** | Ref  **1.91 (1.55-2.34)** | Ref  **1.73 (1.38-2.16)^Household stress, gender, employment^** |
| **ACE score categories**  **None**  **1 ACE**  **2 ACEs**  **3 ACEs**  **4 or more** | Ref  **1.45 (1.14-1.84)**  **1.52 (1.19-1.93)**  **1.70 (1.32-2.18)**  **2.45 (1.97-3.04**) | Ref  **1.33 (1.03-1.71) ^Household stress, gender, employment^**  **1.56 (1.20-2.02)**  **1.56 (1.19-2.05)**  **2.29 (1.80-2.91)** |
| **Level of ACEs**  **None**  **Low**  **High** | Ref  **1.71 (1.40-2.10)**  **2.86 (2.24-3.66)** | Ref  **1.62 (1.29-2.02) ^Household stress, gender, employment^**  **2.53 (1.92-3.33)** |
| ***Dysfunction subscale*** | | |
| **Any ACE**  **No**  **Yes** | Ref  1.09 (0.98-1.22) | Ref  1.04 (0.92-1.17)**^gender, marital status, employment^** |
| **ACE score categories**  **None**  **1 ACE**  **2 ACEs**  **3 ACEs**  **4 or more** | Ref  1.11 (0.97-1.26)  1.06 (0.93-1.21)  1.03 (0.90-1.19)  1.12 (1.00-1.26) | Ref  1.03 (0.90-1.19) **^gender, marital status, employment^**  1.01 (0.88-1.17)  1.00 (0.86-1.17)  1.07 (0.94-1.23) |
| **Level of ACEs**  **None**  **Low**  **High** | Ref  1.07 (0.96-1.20)  **1.16 (1.01-1.33)** | Ref  1.03 (0.91-1.16) **^gender, marital status, employment^**  1.09 (0.93-1.28) |
| ***Depression subscale*** | | |
| **Any ACE**  **No**  **Yes** | Ref  **2.44 (1.74-3.42)** | Ref  **2.00 (1.36-2.97)^Household stress, gender, employment, matric, SES^** |
| **ACE score categories**  **None**  **1 ACE**  **2 ACEs**  **3 ACEs**  **4 or more** | Ref  1.45 (0.98-2.15)  **1.76 (1.18-2.62)**  **2.28 (1.52-3.43)**  **3.39 (2.38-4.84)** | Ref  1.26 (0.81-1.96) **^High household stress, gender, employment, matric^**  **1.63 (1.04-2.54)**  **2.30 (1.45-3.64)**  **2.75 (1.82-4.16)** |
| **Level of ACEs**  **None**  **Low**  **High** | Ref  **2.09 (1.49-2.94)**  **4.13 (2.75-6.20)** | Ref  **1.87 (1.27-2.75) ^High household stress, gender, employment, matric, SES^**  **3.11 (1.94-4.99)** |
