# Supplementary material for: Stress begets stress: the association of adverse childhood experiences with psychological distress in the presence of adult life stress
Source: BMC Public Health. 2018 Jul 5;18:835. doi: 10.1186/s12889-018-5767-0 (PMC6034311; doi:10.1186/s12889-018-5767-0)
Supplement: Supplementary file 5 — Hierarchical regression results: ACEs as binary exposure. Presented here are the adjusted hierarchical regression results using ACE as a binary exposure, including the interaction analysis. (DOCX 14 kb) [file 12889_2018_5767_MOESM5_ESM.docx]

|  | **Model 1** | **Model 2** | **Model 3** | **Model 4 (with interaction)** |
| --- | --- | --- | --- | --- |
| **Chi^2^ Δ** | **77.6*** | **88.3** | **101.4** | **112.8** |
| **Any ACE**  No  Yes | Ref  **3.80 (2.17-6.62)** | Ref  **4.09 (2.14-7.82)** | Ref  **3.32 (1.72-6.40)** | Ref  **2.74 ( 1.03-7.36)** |
| **^Adult stress#Any ACE**  No stress#No ACEs  Low stress#Any ACE  High stress#Any ACE |  |  |  | Ref  2.06 (0.49-8.71)  0.12 ( 0.01-1.21) |
| **Age** | 1.08 (0.85-1.37) | 1.09 (0.84-1.42) | 1.09 (0.83-1.43) | 1.09 (0.83-1.42) |
| **Gender**  Male  Female | Ref  **2.56 (1.95-3.37)** | Ref  **2.67 (1.97-3.61)** | Ref  **2.74 (2.0-13.72)** | Ref  **2.78 (2.04-3.79)** |
| **Marital status**  Single  Relationship | Ref  **0.74 (0.57-0.98)** | Ref  **0.72 (0.53-0.97)** | Ref  **0.71 (0.52-0.96)** | Ref  **0.70 ( 0.51-0.95)** |
| **SES** |  | **0.90 (0.84-0.97)** | **0.90 (0.84-0.97)** | **0.90 ( 0.83-0.97** |
| **Completed matric**  No  Yes |  | Ref  0.77 (0.56-1.06) | Ref  0.79 (0.57-1.09) | Ref  0.79 ( 0.57-1.10) |
| **Employment**  No  Yes |  | Ref  0.79 (0.59-1.07) | Ref  0.82 (0.60-1.10) | Ref  0.81 ( 0.60-1.09) |
| **Adult stress**  None  Low  High |  |  | Ref  **1.96 (1.35-2.85)**  **2.77 (1.70-4.53)** | Ref  0.99 ( 0.25-3.98)  **21.92 ( 2.35-204.86)** |

**Hierarchical regression results: ACEs as binary exposure**

***Bold:** Likelihood ratio chi-square test significant at p<0.05; **^#^Bold OR:** significant at p<0.05; **^**Interaction terms.
